# Association between Clinical Frailty Scale (CFS) and clinical presentation and outcomes in older inpatients with COVID-19

**DOI:** 10.1186/s12877-022-03642-y

**Published:** 2023-01-02

**Authors:** Ana Martí-Pastor, Oscar Moreno-Perez, Esther Lobato-Martínez, Fatima Valero-Sempere, Antonio Amo-Lozano, María-Ángeles Martínez-García, Esperanza Merino, Rosario Sanchez-Martinez, Jose-Manuel Ramos-Rincon

**Affiliations:** 1 Internal Medicine Department, Dr. Balmis General University Hospital, Alicante Institute for Health and Biomedical Research (ISABIAL), Calle Pintor Baeza, 12. CP 03010, Alicante, Spain; 2 Endocrinology and Nutrition Department, Dr. Balmis General University Hospital, Alicante Institute for Health and Biomedical Research (ISABIAL), Alicante, Spain; 3 Clinical Medicine Department, University Miguel Hernández de Elche, Alicante, Spain; 4 Pulmonogy Department, Dr. Balmis General University Hospital, Alicante Institute for Health and Biomedical Research (ISABIAL), Alicante, Spain; 5 Infectious Diseases Unit, Alicante Institute for Health and Biomedical Research (ISABIAL), Dr. Balmis General University Hospital, Alicante, Spain

**Keywords:** COVID-19, Frail elderly, Symptom assessment, Cough, Myalgias, Arthralgias, Anosmia, Dysgeusia, Confusion, Mortality, Patient readmission, Cohort study, Spain

## Abstract

**Background:**

Frailty is a physiological condition characterized by a decreased reserve to stressors. In patients with COVID-19, frailty is a risk factor for in-hospital mortality. The aim of this study was to assess the relationship between clinical presentation, analytical and radiological parameters at admission, and clinical outcomes according to frailty, as defined by the Clinical Frailty Scale (CFS), in old people hospitalized with COVID-19.

**Materials and methods:**

This retrospective cohort study included people aged 65 years and older and admitted with community-acquired COVID-19 from 3 March 2020 to 31 April 2021. Patients were categorized using the CFS. Primary outcomes were symptoms of COVID-19 prior to admission, mortality, readmission, admission in intensive care unit (ICU), and need for invasive mechanical ventilation. Analysis of clinical symptoms, clinical outcomes, and CFS was performed using multivariable logistic regression, and results were expressed as odds ratios (ORs) and 95% confidence intervals (CIs).

**Results:**

Of the 785 included patients, 326 (41.5%, 95% CI 38.1%–45.0%) were defined as frail (CFS ≥ 5 points): 208 (26.5%, 95% CI 23.5%–29.7%) presented mild-moderate frailty (CFS 5–6 points) and 118 (15.0%, 95% CI 12.7%–17.7%), severe frailty (7–9 points). After adjusting for epidemiological variables (age, gender, residence in a nursing home, and Charlson comorbidity index), frail patients were significantly less likely to present dry cough (OR 0.58, 95% CI 0.40–0.83), myalgia-arthralgia (OR 0.46, 95% CI 0.29–0.75), and anosmia-dysgeusia (OR 0.46, 95% CI 0.23-0.94). Confusion was more common in severely frail patients (OR 3.14; 95% CI 1.64-5.97). After adjusting for epidemiological variables, the risk of in-hospital mortality was higher in frail patients (OR 2.79, 95% CI 1.79-4.25), including both those with mild-moderate frailty (OR 1.98, 95% CI 1.23-3.19) and severe frailty (OR 5.44, 95% CI 3.14-9.42). Readmission was higher in frail patients (OR 2.11, 95% CI 1.07–4.16), but only in mild-moderate frailty (OR 2.35, 95% CI 1.17–4.75)..

**Conclusion:**

Frail patients presented atypical symptoms (less dry cough, myalgia-arthralgia, and anosmia-dysgeusia, and more confusion). Frailty was an independent predictor for death, regardless of severity, and mild-moderate frailty was associated with readmission.

**Supplementary Information:**

The online version contains supplementary material available at 10.1186/s12877-022-03642-y.

## ‘What’s known?’ and ‘What’s new?’

- The Clinical Frailty Scale is an easy measure of frailty used in COVID-19 and a risk factor for in-hospital mortality.
- We analyzed the clinical presentation and outcomes of COVID-19 according to frailty.
- Frailty is associated with atypical clinical presentation.
- Frailty is an independent predictor for death and readmission.

## Introduction

The global COVID-19 pandemic, caused by severe acute respiratory distress syndrome coronavirus 2 (SARS-CoV2), has caused more than 554 million known infections and well over 6.3 million deaths globally as of 9 July 2022 [1]. Over two years into the pandemic, COVID-19 continues to cause high mortality and hospitalization rates in older adults [2, 3].

Frailty is a clinical condition or state characterized by a decreased reserve to stressors [4, 5], associated with healthcare-related outcomes such as disability, hospitalization, and death [4, 6–8]. It can be measured with different instruments, the most common of which is the Clinical Frailty Scale (CSF) [9]. Several COVID-19 studies have pointed to frailty as an independent predictor for a fatal outcome [10–13]. Other authors have proposed screening for frailty to inform early decision-making on resource allocation and clinical management [4].

Chronic diseases and other health deficits tend to be more common in frail people, and these can obscure the recognition of new symptoms [14]. Several studies have reported an association between frailty and atypical clinical presentation of infectious diseases [15]. However, it is unclear whether frailty may also be a predictor for atypical symptoms in older patients with COVID-19 [11, 15].

The aim of this study was to assess patient characteristics on admission (clinical, analytical and radiological features) and clinical outcomes (mortality, readmission, admission in intensive care unit [ICU], and need for invasive mechanical ventilation [IMV]) according to CFS-assessed frailty [9] in hospitalized patients aged 65 years or older with COVID-19. We hypothesized that frailty would be strongly related with atypical symptoms and clinical outcomes.

## Materials and methods

### Study design and population

This retrospective cohort study took place from 3 March 2020 to 31 April 2021 at the Dr. Balmis General University Hospital (Alicante, Spain). Eligible patients were adults (≥ 65 years) admitted to hospital and diagnosed with COVID-19 pneumonia using the reverse transcriptase polymerase chain reaction (RT-PCR) test for SARS-CoV-2. Patients under 65 years old, those with nosocomial acquisition, and those who had been vaccinated were excluded.

### Frailty assessment

Frailty was assessed using the CFS, which is based on the patient’s condition two weeks prior to hospital admission.9 Patients are scored on an ordinal scale from 1 to 9, with the score of 1 indicating that the person is very fit; 2 well; 3 managing well; 4 vulnerable; 5 mildly frail; 6 moderately frail; 7 severely frail; 8 very severely frail; and 9 terminally ill [9, 16]. A single geriatrician retrospectively decided the degree of frailty in all patients by scoring the CFS and combining information from inpatient and outpatient electronic medical records. All borderline cases were adjudicated by another specialist physician in line with our previous study [17]  Both physicians were blinded to clinical outcomes.

We did not anticipate an adequate number of events for each score, so we grouped them as 1–4 (no frailty), 5–6 (mild-moderate frailty, with initial signs of frailty but some degree of independence), and 7–9 (severe frailty) for the purposes of the analyses. We also analyzed CFS as a dichotomous variable (no frailty [1–4] versus frailty [5–9]).

### Variables and data collection

More in-depth information about the data collection and definition of variables has been provided in papers by the COVID-19 ALC research group [18, 19]. Pre-admission comorbidities were collected from the patient’s electronic medical record. The burden of comorbidities was assessed using the age-adjusted Charlson comorbidity index (CCI), a method of estimating mortality risk from comorbid disease and two-year mortality at a specified time point in longitudinal studies [20].

On admission, laboratory data were collected (total lymphocytes, C-reactive protein, procalcitonin, ferritin, lactate dehydrogenase, D-dimer, interleukin-6, brain natriuretic peptide (BNP), potassium, estimated glomerular filtration rate [eGFR] according to the CKD-EPI equation, troponin). Opacities on chest X-ray were also assessed.

The main outcome variables were: (A) clinical presentation (symptoms), including duration of symptoms prior to admission, fever, dyspnea, dry cough, wet cough, asthenia, diarrhea, myalgia-arthralgia, confusion and anosmia-dysgeusia; and (B) clinical outcomes, including all in-hospital mortality, readmission, admission in ICU, and need for IMV. Confusion was defined according to the Glasgow Coma Scale as a score of less than 14 points. Asthenia was considered present if the patient or their relatives reported weakness or lack of energy and strength. In cases of patients with altered mental status, some conditions, including asthenia, confusion, myalgia-arthralgia, and anosmia-dysgeusia were evaluated using the medical history as related by relatives or caregiver.

### Statistical analysis

Categorical variables are expressed as frequencies (percentages) and continuous variables as medians (interquartile range, IQR) or mean (standard deviation). Differences between groups were analyzed using the Mann-Whitney U test for continuous variables and Pearson’s chi-square test for categorical variables. Differences in CFS as dichotomous (no frailty versus frailty) and categorical (no frailty, mild-moderate frailty, and severe frailty) variables were calculated using odds ratios (ORs) with 95% confidence intervals (CIs).

Multivariable logistic regression models were fit to study the association of frailty with initial clinical features and clinical outcomes. In model A, analyses were adjusted for epidemiological variables including age group (65-74 years, 75-84 years, and $\geq$  85 years), sex (male/female), residence in nursing home (yes/no), and CCI (0-4 and $\geq$ 5 points). The analysis of clinical outcomes was additionally adjusted for analytical variables yielding statistically significant results in the bivariable analysis (model B), including: procalcitonin > 0.5 ng/mL, ferritin > 500 mg/L, lactate dehydrogenase > 250 U/L, D-dimers > 1 mg/mL, eGFR < 60 ml/min/m2, troponin T > 14 ng/L, and brain natriuretic peptide > 125 pg/mL

The log-likelihood function was used to determine whether one model fit the data better than another. The log-likelihood ensures that the maximum value of the log probability occurs at the same point as the original probability function. Moreover, to evaluate the goodness-of-fit of the logistic regression model, we used the Cox-Snell R2 and Nagelkerke R2 statistics. The Nagelkerke R2 test is an adjusted version of the Cox-Snell R2 (Supplementary Table 1-3). Both tests measure the proportion of the total variation of the dependent variable that can be explained by independent variables in the current model. The Cox-Snell R2 and Nagelkerke R2 express the explanatory power of the model. All analyses were performed using IBM SPSS Statistics for Windows, Version 25.0 (Armonk, NY IBM Corp). Statistical significance was set at p < 0.05.

### Ethical aspects

This work was approved by the Institutional Research Ethics of Dr. Balmis General University Hospital – ISABIAL (EXP. 200145). The requirement to obtain informed consent from participants was waived by the Institutional Research Ethics of Dr. Balmis General University Hospital – ISABIAL. The research was conducted according to the principles of the Declaration of Helsinki.

## Results

A total of 1745 adults with PCR-confirmed COVID-19 were admitted to Dr. Balmis General University Hospital during the study period. Of these, we excluded 63 patients with a nosocomial infection, 840 who were under 65 years of age, and 55 who were fully vaccinated, leaving a sample of 785.

Table 1 shows the number of patients according to their CSF score. Mean CFS was 4.6 (standard deviation 1.6), and 326 (41.5 %, 95% CI 38.1%–45.0%) patients were defined as frail (CFS ≥ 5 points). Frailty was mild-moderate (5–6 points) in 208 (26.5%, 95% CI 23.5%–29.7%) and severe (7–9 points) in 118 (15.0%, 95% CI 12.7%–17.7%). Prevalence of frailty increased with age: in patients aged 65-74 years, it was 13.8%; 75-84 years, 40.5%; and 85 years or older, 45.7% (*p* < 0.001). Table 1 shows demographics, comorbidities, laboratory findings, and outcomes according to CFS.

**Table 1:** Number of patients aged ≥ 65 years with confirmed COVID-19, according to Clinical Frailty Scale

Clinical Frailty Scale	N (%)
1	0
2	27 (3.4)
3	214 (27.3)
4	218 (27.8)
5	98 (12.5)
6	110 (14.0)
7	77 (9.8)
8	28 (3.6)
9	13 (1.7)

### Clinical presentation and frailty

The median duration of symptoms before admission was seven days in non-frail patients, five days in those with mild-moderate frailty, and three days in severely frail patients (*p*<0.001). In the bivariable analysis, confusion was associated with frailty (no frailty, 7.6%; mild-moderate frailty, 16.7%; and severe frailty, 29.2%; *p* < 0.001, Table 2). Fever, dry cough, asthenia, diarrhea, myalgias-arthralgia, and anosmia-dysgeusia were significantly less common in patients with frailty, regardless of severity (Table 2). Dyspnea and wet cough were similar between groups.

**Table 2:** Epidemiology, clinical presentation, initial analytical and radiological assessment, and clinical outcomes in patients ≥ 65 years with confirmed COVID-19, according to Clinical Frailty Score

	N	Total sample	Non-frail (1–4) (*n* = 459)	Mild- moderate frailty (5–6) (*n* = 208)	Severe frailty (7–9) (*n* = 118)	*P* value	Frail (5–9) (*n* = 326)	Non-frail vs. frail *P* value
**Epidemiological variables**								
Age, years, median (IQR)	785	78 (71–84)	74 (69–79)	83 (79–88)	85 (81–90)	**< 0.001**	84 (80–88)	**< 0.001**
Age group, n (%)	785					**< 0.001**		**< 0.001**
65–74 years		301 (38.3)	256 (55.8)	32 (15.4)	13 (11.0)		45 (13.8)	
75–84 years		291 (37.1)	159 (34.6)	88 (42.3)	44 (37.3)		132 (40.5)	
85–100 years		291 (37.1)	44 (9.6)	88 (42.3)	44 (37.3)		149 (45.7)	
Female sex, n (%)	785	364 (46.4)	192 (41.8)	106 (51.0)	66 (55.9)	**0.007**	172 (52.8)	0.002
First wave, n (%)	785	142 (18.1)	94 (20.5)	30 (14.4)	18 (15.3)	0.11	48 (14.7)	0.039
Long-term care resident, n (%)	785	52 (6.6)	6 (1.3)	16 (7.7)	30 (25.4)	**< 0.001**	46 (14.1)	**< 0.001**
CCI, median (IQR)	781	5 (4–7)	4 (3–5)	6 (5–9)	7 (6–9)	< 0.001	7 (5–8)	**< 0.001**
Baseline CCI ≥ 5, n (%)	781	458 (58.8)	177 (38.6)	172 (83.5)	109 (94.0)	**< 0.001**	281 (86.1)	**< 0.001**
**Clinical presentation**								
Duration of symptoms (days), median (IQR)	743	6 (3–9)	7 (4–10)	5 (2–9)	3 (1–7)	**< 0.001**	4 (2–8)	**< 0.001**
Duration of symptoms ≤ 5 days, n (%)	743	340 (45.6)	175 (38.8)	97 (50.0)	68 (67.3)	**< 0001**	165 (55.9)	**< 0.001**
Fever, n (%)	778	387 (52.7)	280 (61.1)	93 (45.1)	52 (45.6)	**< 0.001**	145 (45.3)	**< 0.001**
Dyspnea, n (%)	782	453(57.9)	263 (57.3)	127 (61.1)	63 (54.8)	0.50	190 (58.8)	0.49
Dry cough, n (%)	777	371 (47.7)	249 (54.4)	89 (43.2)	33 (29.2)	**< 0.001**	122 (38.2)	**< 0.001**
Asthenia, n (%)	771	361 (46.8)	234 (51.5)	87 (42.6)	40 (35.4)	**0.003**	127 (46.8)	**0.002**
Diarrhea, n (%)	772	182 (23.6)	123 (27.0)	48 (23.4)	11 (9.8)	**0.001**	59 (18.6)	**0.007**
Myalgia-arthralgia, n (%)	767	171 (22.3)	136 (29.9)	27 (13.4)	8 (7.3)	**< 0.001**	35 (11.2)	**< 0.001**
Wet cough, n (%)	777	133 (17.1)	78 (17.0)	34 (16.5)	21 (18.6)	0.79	55 (17.2)	0.92
Confusion, n (%)	779	100 (12.8)	35 (7.6)	32 (16.7)	33 (29.2)	**< 0.001**	65 (20.3)	**< 0.001**
Anosmia-dysgeusia, n (%)	762	78 (10.2)	64 (14.2)	12 (6.0)	2 (1.8)	**< 0.001**	14 (4.5)	**< 0.001**
**Analytical and radiological parameters on admission**								
Oximetry < 94% at room temperature, n (%)	735	349 (47.3)	209 (47.9)	89 (46.1)	50 (47.2)	0.82	139 (47.3)	0.66
Lymphocytes < 1.0 × 10^3^/L, n (%)	785	451 (57.5)	260 (56.6)	121 (58.2)	70 (59.3)	0.85	191 (58.9)	0.92
C-reactive protein > 10 mg/dL, n (%)	782	284 (36.3)	173 (37.8)	69 (33.3)	42 (35.9)	0.215	111 (34.3)	0.31
Procalcitonin > 0.5 ng/mL, n (%)	733	106 (14.5)	48 (11.2)	31 16.3)	27 (23.9)	**0.002**	58 (19.1)	**0.002**
Ferritin > 500 mg/L, n (%)	725	412 (56.8)	270 (63.2)	92 (46.7)	50 (49.5)	**< 0.001**	142 (47.7)	**< 0.001**
Lactate dehydrogenase > 250 U/L, n (%)	735	487 (66.3)	305 (70.6)	118 (60.8)	64 (58.7)	**0.011**	182 (60.1)	**0.003**
D-dimers > 1 mg/mL, n (%)	735	357 (51.4)	171 (39.6)	103 (52.3)	83 (78.3)	**< 0.001**	186 (61.4)	**< 0.001**
Interleukin 6 > 10 pg/mL, n (%)	587	471 (80.2)	279 (79.1)	131 (83.4)	61 (76.3)	0.39	192 (81.0)	0.92
Brain natriuretic peptide > 125 pg/mL, n (%)	722	591 (81.9)	309 (73.5)	175 (91.5)	108 (96.4)	**< 0.001**	283 (93.4)	**< 0.001**
Potassium < 3.5 mmol/L, n (%)	639	78 (12.2)	51 (14.0)	20 (11.3)	7 (7.1)	0.053	27 (9.8)	0.11
eGFR < 60 ml/min/m^2^, n (%)	781	375 (48.0)	171 (37.5)	125 (60.1)	79 (67.5)	**< 0.001**	204 (62.8)	**< 0.001**
Troponin T > 14 ng/L, n (%)	731	489 (66.9)	225 (52.4)	160 (83.3)	104 (94.5)	**< 0.001**	264 (87.4)	**< 0.001**
Opacities > 50% of lung surface on X-rays, n (%)	785	196 (25.0)	121 (26.4)	54 (26.0)	21 (17.8)	0.13	75 (23.0)	0.20
**Clinical outcomes**								
In-hospital mortality, n (%)	785	178 (22.7)	58 (12.6)	57 (27.4)	63 (53.4)	**< 0.001**	120 (36.6)	**< 0.001**
Readmission, n (%)	785	55 (7.2)	21 (4.8)	25 (12.1)	9 (8.0)	**0.003**	34 (10.7)	**0.002**
Intensive care unit admission, n (%)	784	114 (14.4)	99 (21.6)	14 (6.7)	1 (0.9)	**< 0.001**	15 (4.6)	**< 0.001**
Invasive mechanical ventilation, n (%)	781	78 (10.0)	67 (14.8)	11 (5.3)	0 (0.0)	**< 0.001**	11 (3.4)	**< 0.001**
Length of hospital stay (days), median (IQR)	774	10 (6–16)	9 (6–15)	10 (6–18)	11 (7–15)	0.36	10 (6–17)	0.16
Length of hospital stay < 10 days, n (%)	774	372 (48.1)	228 (50.4)	94 (45.2)	50 (43.9)	0.28	144 (44.7)	0.12

In bold, significantly statistically values

*CCI* Charlson comorbidity index, *eGFR* estimated glomerular filtration rate, *IQR* interquartile range

After adjusting for epidemiological variables, the multivariable analysis (Table 3; supplementary table 1) showed that frail patients had a lower risk of dry cough (OR 0.58, 95% CI 0.40–0.83) and myalgia-arthralgia (OR 0.46, 95% CI 0.29–0.75). This was true for patients with both mild-moderate frailty (OR 0.66, 95% CI 0.45–0.97; OR 0.53, 95% CI 0.32–0.88, respectively) and severe frailty (OR 0.40, 95% CI 0.23–0.67; OR 0.30, 95% CI 0.13–0.70, respectively). Anosmia-dysgeusia were also significantly less common in frail patients (OR 0.46, 95% CI 0.23–0.94), especially in  severely frail cases (OR 0.21, 95% CI 0.04–0.95). In contrast, confusion was significantly more common in frail patients (OR 2.01, 95% CI 1.18–3.43), which is also attributable to the severe frailty subgroup (OR 3.14, 95% CI 1.64–5.97). Finally, a duration of symptoms of five days or less prior to admission was more common in patients with severe frailty (OR 1.99, 95% CI 1.17–3.28).

**Table 3:** Bivariable and multivariable logistic regression analysis of symptoms according to Clinical Frailty Score (reference value: no frailty)

Symptoms according to frailty	Crude OR (95% CI)	*P* value	Adjusted OR (95% CI)^a^	*P* value
**Duration of symptoms < 5 days prior to admission**				
Mild-moderate frailty	1.57 (1.12–2.21)	**0.008**	1.11 (0.74–1.64)	0.59
Severe frailty	3.25 (1.05–5.13)	**< 0.001**	1.99 (1.17–3.28)	**0.010**
Frailty	2.00 (1.49–2.69)	**< 0.001**	1.30 (0.90–1.87)	0.20
**Fever**				
Mild-moderate frailty	0.52 (0.37–0.73)	**< 0.001**	0.78 (0.53–1.15)	0.22
Severe frailty	0.3 (0.35–0.81)	**0.003**	0.84 (0.51–1.38)	0.56
Frailty	0.52 (0.39–0.70)	**< 0.001**	0.51 (0.80–1.11)	0.50
**Dry cough**				
Mild-moderate frailty	0.63 (0.46–0.88)	**< 0.001**	0.66 (0.45–0.97)	**0.037**
Severe frailty	0.35 (0.22–0.54)	**< 0.001**	0.40 (0.23–0.67)	**0.001**
Frailty	0.52 (0.39–0.70)	**< 0.001**	0.58 (0.40–0.83)	**0.003**
**Wet cough**				
Mild-moderate frailty	0.96 (0.61–1.49)	0.86	0.79 (0.47–1.33)	0.37
Severe frailty	1.11 (0.65–1.49)	0.70	0.82 (0.43–1.55)	0.53
Frailty	1.01 (0.70–1.48)	0.92	0.50 (0.81–1.27)	0.35
**Dyspnea**				
Mild-moderate frailty	1.10 (0.73–1.67)	0.63	1.39 (0.84–2.28)	0.19
Severe frailty	1.29 (0.81–2.055)	0.27	1.50 (0.92–2.44)	0.10
Frailty	0.93 (0.70–1.25)	0.67	1.04 (0.73–1.49)	0.81
**Anosmia-dysgeusia**				
Mild-moderate frailty	0.38 (0.20–0.73)	**0.003**	0.56 (0.27–1.15)	0.12
Severe frailty	0.11 (0.03–0.47)	**0.003**	0.21 (0.04–0.95)	**0.044**
Frailty	0.29 (0.16–0.52)	**< 0.001**	0.46 (0.23–0.94)	**0.033**
**Myalgia-arthralgia**				
Mild-moderate frailty	0.36 (0.23–0.56)	**< 0.001**	0.53 (0.32–0.88)	**0.014**
Severe frailty	0.18 (0.09–0.39)	**< 0.001**	0.30 (0.13–0.70)	**0.005**
Frailty	0.29 (0.19–0.44)	**< 0.001**	0.46 (0.29–0.75)	**0.002**
**Asthenia**				
Mild-moderate frailty	0.69 (0.50–0.97)	**0.035**	0.80 (0.55–1.16)	0.23
Severe frailty	0.51 (0.33–0.79)	**0.002**	0.70 (0.42–1.16)	0.17
Frailty	0.62 (0.47–0.84)	**0.002**	0.73 (0.54–1.10)	0.16
**Diarrhea**				
Mild-moderate frailty	0.82 (0.56–1.21)	0.33	1.25 (0.79–1.97)	0.34
Severe frailty	0.29 (0.15–0.56)	**< 0.001**	0.52 (0.27–1.18)	0.13
Frailty	0.61 (0.43–0.87)	**0.007**	1.05 (0.68–1.62)	0.83
**Confusion**				
Mild-moderate frailty	2.21 (1.32–3.69)	**0.002**	1.59 (0.88–2.85)	0.16
Severe frailty	4.99 (2.93–8.50)	**< 0.001**	3.14 (1.64–5.97)	**< 0.001**
Frailty	3.02 (1.99–4.78)	**< 0.001**	2.01 (1.18–3.43)	**0.010**

Clinical Frailty Score: 0–4, no frailty; 5–9, any level of frailty; 5–6, mild-moderate frailty; 7–9, severe frailty

In bold, significantly statistically values

^a^Adjusted for age group (65–74, 75–84 and ≥85 years), sex, wave of infections, residence in nursing home, Charlson comorbidity index (dichotomized: 0–4 and ≥ 5)

## Analytical and radiological parameters and frailty

Regarding analytical and radiological parameters on admission, bivariable analysis showed that frail patients (both mild-moderate and severe frailty) were more likely to show procalcitonin > 0.5 ng, D-dimers > 1 mg/mL, grain natriuretic peptide > 125 pg/mL, eGFR < 60 ml/min/m^2^, and troponin T > 14 ng/L, along with a lower eGFR. Compared to non-frail patients, a smaller proportion of frail patients had ferritin > 500 mg/L and lactate dehydrogenase > 250 U/L.  Parameters showing no relationship with frailty were lymphocytes, C-reactive protein, interleukin 6, potassium, and opacities on more than 50% of lung surface on chest X-ray (Table 1).

After adjusting for epidemiological variables, frailty (both mild-moderate and severe) remained significantly associated with elevated BNP (frail: OR 2.23, 95% 1.23–4.05; mild-moderate frailty: OR 1.97, 95% CI 1.05–3.71; severe frailty: OR 3.51, 95% CI 1.11–11.04) and troponin T (frail: OR 2.70, 95% 1.69–4.32; mild-moderate frailty: OR 2.28, 95% CI 1.39–3.75; severe frailty: OR 5.12, 95% CI 2.04–12.86) on admission. The association with high procalcitonin and D-dimers held only for patients with severe frailty (OR 2.02, 95% CI 1.06–3.84; OR 4.27, 95% CI 2.44–7.84, respectively; Table 4, supplementary Table 2).

**Table 4:** Bivariable and multivariable logistic regression analyses of analytical and radiological parameters on admission, according to Clinical Frailty Score (reference value: no frailty)

Analytical and radiological variables	Frailty	Crude OR (95% CI)	*P* value	Adjusted OR (95% CI)^a^	*P* value
Oximetry < 94% at room temperature	Mild-moderate	0.93 (0.66–1.35)	0.70	0.98 (0.66–1.43)	0.928
	Severe	0.97 (0.63–1.48)	0.89	0.99 (0.60–1.64)	> 0.99
	Any frailty	0.94 (0.70–1.26)	0.70	0.98 (0.68–1.42)	0.94
Lymphocytes < 1.0 × 10^3^/L	Mild-moderate	1.06 (0.67–1.48)	0.71	0.78 (0.59–1.29)	0.52
	Severe	1.11 (0.74–1.68)	0.60	1.00 (0.60–1.64)	> 0.99
	Any frailty	1.08 (0.82–1.44)	0.59	0.91 (0.63–1.32)	0.91
C-reactive protein > 10 mg/dL	Mild-moderate	0.24 (0.58–1.16)	0.27	0.86 (0.58–1.29)	0.49
	Severe	0.92 (0.60–1.40)	0.78	0.97 (0.58–1.62)	0.91
	Any frailty	0.85 (0.63–1.15)	0.65	0.89 (0.61–1.34)	0.57
Procalcitonin > 0.5 ng/mL	Mild-moderate	1.55 (0.95–2.52)	0.078	1.35 (0.77–2.36)	0.29
	Severe	2.49 (1.47–4.22)	**0.001**	2.02 (1.06–3.84)	**0.032**
	Any frailty	1.88 (1.24–2.85)	**0.003**	1.54 (0.93–2.52)	0.092
Ferritin > 500 mg/L	Mild-moderate	0.51 (0.36–0.71)	**< 0.001**	0.78 (0.52–1.17)	0.24
	Severe	0.57 (0.37–0.88)	**0.012**	0.98 (0.57–1.67)	0.23
	Any frailty	0.59 (0.39–0.71)	**< 0.001**	0.83 (0.57–1.21)	0.35
Lactate dehydrogenase > 250 U/L	Mild-moderate	0.64 (0.45–0.92)	**0.016**	0.73 (0.49–1.18)	0.73
	Severe	0.59 (0.38–0.91)	**0.018**	0.71 (0.46–1.20)	0.72
	Any frailty	0.62 (0.46–0.58)	**0.003**	0.73 (0.50–1.07)	0.11
D-dimers > 1 mg/mL	Mild-moderate	1.67 (1.19–2.34)	**0.003**	1.29 (0.87–1.92)	0.19
	Severe	5.01 (3.33–9.8)	**< 0.001**	4.27 (2.44–7.84)	**< 0.001**
	Any frailty	2.24 (1.79–3.27)	**< 0.001**	1.76 (1.22–2.54)	**0.003**
Interleukin 6 > 10 pg/mL	Mild-moderate	1.29 (0.78–2.109	0.35	1.3 (0.82–2.54)	0.18
	Severe	0.86 (0.45–1.45)	0.49	0.80 (0.40–1.61)	0.54
	Any frailty	01.08 (0.71–1.64)	0.70	1.22 (0.73–2.04)	0.43
Brain natriuretic peptide > 125 pg/mL	Mild-moderate	3.94 (2.26–6.87)	**< 0.001**	1.97 (1.05–3.71)	**0.034**
	Severe	0.97 (3.50–27.1)	**< 0.001**	3.51 (1.11–11.04)	**0.031**
	Any frailty	5 0.10 (3.08–8.43)	**< 0.001**	2.23 (1.23–4.05)	**0.005**
Potassium < 3.5 mmol/L	Mild-moderate	0.78 (0.45–1.35)	0.38	0.78 (0.48–1.74)	0.79
	Severe	0.46 (0.20–1.06)	**0.069**	0.46 (0.17–1.28)	0.14
	Any frailty	0.66 (0.40–1.09)	0.10	0.78 (0.43–1.46)	0.45
eGFR < 60 ml/min/m^2^	Mild-moderate	2.51 (1.79–3.51)	**< 0.001**	1.30 (0.87–1.93)	0.19
	Severe	3.46 (2.25–5.33)	**< 0.001**	1.62 (0.96–2.73)	0.065
	Any frailty	2.81 (2.09–3.71)	**< 0.001**	1.48 (0.96-2.00)	0.079
Troponin T > 14 ng/L	Mild-moderate	4.53 (2.96–6.92)	**< 0.001**	2.28 (1.39–3.75)	**0,001**
	Severe	15.71 (6.75–36.5)	**< 0.001**	5.12 (2.04–12.86)	**0.001**
	Any frailty	6.29 (4.26–2.29)	**< 0.001**	2.70 (1.69–4.32)	**< 0.001**
Opacities > 50% of lung surface on X-rays	Mild-moderate	0.97 (0.67–1.42)	0.91	0.95 (0.61–1.49)	0.084
	Severe	0.60 (0.36–1.10)	0.056	0.58 (0.31–1.08)	0.090
	Any frailty	0.83 (0.59–1.16)	0.29	0.39 (0.55–1.29)	0.42

Clinical Frailty Score: 0–4, no frailty; 5–9, any level of frailty; 5–6, mild-moderate frailty; 7–9, severe frailty

In bold, significantly statistically values

^a^Adjusted for age group (65–74, 75–84 and ≥85 years), sex, wave, residence in nursing home, Charlson comorbidity index (dichotomized 0–4 and ≥ 5)

## Clinical outcomes and frailty

In the bivariable analysis, in-hospital mortality was associated with frailty: 33.6% of all frail patients died (mild-moderate 27.4%, severe 53.4%), compared to 12.6% non-frail patients (*p* < 0.001, Table 1). Readmission was also higher in frail patients (frail: 10.7% (mild-moderate frailty: 12.1%, severe frailty: 8.0%) compared to the non-frail group (4.8%, *p* = 0.003, Table 1). On the other hand, admission to the ICU and need for IMV was higher in non-frail patients (21.6% and 14.8%, respectively) compared to those with mild-moderate frailty (6.7% and 5.3%) or severe frailty (0.9% and 0.0%) (*p* < 0.001, Table 1).

After adjusting for epidemiological and analytical variables (model B), frail patients were more likely than non-frail patients to die during admission (frail: OR 3.74, 95% CI 2.19-6.38; mild-moderate frailty: OR 2.74, 95% CI 1.54-4.49; and severe frailty: OR 9.28, 95% CI 4.52-18.87). Readmission was higher in mild-moderate frailty (model A: OR 2.35, 95% CI 1.17-4.75 and model B: OR 2.35, 95% CI 1.07-5.14) (Table 5, Regardless of the multivariable model used (A or B), admission to the ICU and the need for IMV were similar in non-frail and frail patients (Table 5).

**Table 5:** Bivariable and multivariable logistic regression analyses of clinical outcomes, according to Clinical Frailty Score (reference value: no frailty)

Clinical outcomes according to frailty	Crude OR (95% CI)	*P* value	Model A Adjusted OR (95% CIs)^a^	*P* value	Model B Adjusted OR (95% CI)^b^	*P* value
**In-hospital mortality**						
Mild-moderate frailty	2.26 (1.73–3.93)	**< 0.001**	1.98 (1.23–3.19)	**0.005**	2.74 (1.54–4.89)	**< 0.001**
Severe frailty	7.19 (5.02–12.47)	**< 0.001**	5.44 (3.14–9.42)	**< 0.001**	9.28 (4.52–18.87)	**< 0.001**
Frailty	4.03 (2.82–5.72)	**< 0.001**	2.79 (1.79–4.25)	**< 0.001**	3.74 (2.19–6.38)	**< 0.001**
**Admission in intensive care unit**						
Mild-moderate frailty	0.26 (0.14–4.72)	**< 0.001**	0.72 (0.37–1.40)	0.34	0.65 (0.29–1.45)	0.30
Severe frailty	0.031 (0.00-0.23)	**0.001**	0.14 (0.19–1.08)	0.059	0.16 (0.02–1.39)	0.09
Frailty	0.17 (0.10-0-31)	**< 0.001**	0.59 (0.30–1.10)	0.096	0.53 (0.24–1.16)	0.12
**Invasive mechanical ventilation**						
Mild-moderate frailty	0.33 (0.17–0.69)	**< 0.001**	0.95 (0.45–2.10)	0.90	0.97 (0.41–2.28)	0.95
Severe frailty	NA	NA	NA	NA	NA	NA
Frailty	0.21 (0.10–0.39)	**< 0.001**	0.70 (0.33–1.49)	0.91	0.71 (0.30–1.69)	0.45
**Readmission**						
Mild-moderate frailty	2.76 (1.50–5.06)	**0.001**	2.35 (1.17–4.75)	**0.016**	2.35 (1.07–5.14)	**0.032**
Severe frailty	1.73 (0.77–3.89)	0.18	1.53 (0.61–3.81)	0.36	0.85 (0.25–2.79)	0.79
Frailty	2.28 (1.35–4.19)	**0.003**	2.11 (1.07–4.16)	**0.031**	1.94 (0.88–4.25)	0.098

Clinical Frailty Score: 0–4, no frailty; 5–9, any level of frailty; 5–6, mild-moderate frailty; 7–9, severe frailty

*NA* not available, *OR* odds ratio, *CI* confidence interval

In bold, significantly statistically values

^a^Model A adjusted for age group (65–74, 75–84 and ≥85 years), sex, wave, residence in nursing home, Charlson comorbidity index (dichotomized 0–4 and ≥ 5)

^b^Model B adjusted for variables in model A, plus procalcitonin > 0.5 ng/mL, ferritin > 500 mg/L, lactate dehydrogenase > 250 U/L, brain natriuretic peptide > 125 pg/mL D-dimers > 1 mg/mL, estimated glomerular filtration rate < 60 ml/min/m2, troponin T > 14 ng/L

## Discussion

This study analyzed the association between frailty according to the CFS and clinical presentation, analytical and radiological parameters, and clinical outcomes in older inpatients with COVID-19. Our results show that frailty is independently associated with an atypical presentation, elevated BNP and troponin T, in-hospital mortality, and readmission.

The prevalence of frailty in people with COVID-19 depends on the scale used and the age of the sample population. Polo et al. [15] reported a prevalence of frailty of 25% in patients aged over 50 years, Tehnari et al. [21] of 50% in patients over 65, and Collins et al. [22] of 66.9% in patients over 65. In our study, 41.5% of the cohort aged 65 or older met CFS criteria for frailty, which is consistent with previous research.

The scant literature on the topic suggests that frailty is associated with atypical clinical presentation in infectious disease [15, 23, 24]. Typical features of COVID-19, like fever and cough, were less prevalent in frail older patients, as reported elsewhere [15, 23], although in our multivariable analysis fever did not show significant, independent differences according to frailty. Dry cough presented less frequently in frail patients, as reported by Poco et al. [15] and Jachymek et al., [24] and this difference was significant after adjusting for confounders.  However, our results contrast with other studies showing that this symptom is more common in older, frail patients with COVID-19 [15, 23, 25] 

Myalgia-arthralgia was less common in frail patients in multivariable analysis, contradicting other results showing no association [24]. Anosmia-dysgeusia was likewise less common in frail patients, independently of other variables. It is well known that the prevalence of anosmia-dysgeusia decreases with the age of infected patients, as von Bartheld et al. concluded from their meta-analysis. [23] The reduced presence of this symptom in frail patients has been described in fewer studies [24].

Confusion was more prevalent in frail—especially severely frail—patients.This result is consistent with the reduced level of consciousness and higher rates of delirium and confusion reported by other authors [15, 23]. Finally, asthenia, wet cough, and diarrhea were not significantly different after adjusting for epidemiological and analytical variables in frail versus non-frail patients.

Atypical presentation on emergency admission due to COVID-19 in frail older adults may be attributable to several factors. Frailty and chronic inflammation are linked to immunosenescence and can influence response to infection and subsequent immunity [23, 26]. In frail older patients, changes in innate immunity and a defective activation pathway, possibly due to desensitization and a reduced antibody response to vaccines, have already been linked to atypical symptoms [27]. Moreover, older age and frailty are also associated with a higher CCI, which can impair the ability to recognize and report symptoms of the acute disease [25, 26].

After adjusting for epidemiological variables, frail patients were more likely to have elevated BNP and troponin T levels on admission. Moreover, severely frail patients were more likely than non-frail patients to have high procalcitonin and D-dimers. These associations may be attributable to a higher CCI in frail patients, with more cardiovascular comorbidities and concomitant bacterial infections, not specifically assessed in this study. However, we did not observe an association between frailty and markers of inflammation like C-reactive protein, ferritin, and lactate dehydrogenase.

Numerous studies show that frailty is independently associated with mortality, and several meta-analyses have demonstrated that CFS is positively correlated with mortality [28, 29], especially in people over 65 years of age [15, 16, 21, 30, 31]. In our study, mortality was independently correlated with the level of frailty. In addition, evidence suggests that clinical frailty is determined more by the number of dysfunctional organ systems than by abnormalities in any system in particular [29, 32]

Use of life-sustaining therapies (ICU admission, IMV) was more limited in patients with CFS-defined frailty, but after adjusting for epidemiological and analytical variables, these differences were not significant. These finding are similar to reports from a recent meta-analysis of frailty in COVID-19.30 In our study, readmission was also higher in frail patients, especially in those with mild-moderate frailty. Previous studies have noted a higher risk of readmission in frail individuals with multiple chronic diseases after surgical or medical admission [33]. In our study the patients with severe frailty did not have a higher risk of readmission. This may be because severely frail people are more likely to already be under constant and closely supervised care, whether at home or in a nursing home, and in some cases they die in these settings. However, this question was beyond the scope of this study.

Our results should be interpreted in light of the study’s limitations. Frailty was evaluated retrospectively, so calculation of the CFS relied on information obtained from electronic health records [21], which may have resulted in an underestimation of prevalence. However, several published studies also used retrospective methods for assessing the CFS [15, 21]. Furthermore, this is a single-center study, so caution is warranted when extrapolating our results to other healthcare settings. Finally, we do not have data on the variants of SARS-CoV-2 in patients hospitalized with COVID-19 and cannot rule out a reduction in complications and mortality after the first wave.

## Conclusion

In this study of COVID-19 in older inpatients in a university hospital in Spain, CFS-defined frailty was independently associated with atypical clinical presentation on admission, with less dry cough, myalgia-arthralgia, and anosmia-dysgeusia, and more confusion after adjusting for confounders. Frailty was an independent prognostic factor for death, while mild-moderate frailty was related to readmission. Further multicenter and prospective studies are necessary to understand the real relevance of frailty in the clinical presentation in old people with COVID-19.

## Supplementary Information

**Additional file 1: Supplementary Table 1**. Summary of the models used in the multivariable logistic regression analysis of symptoms according to Clinical Frailty Scale. **Supplementary Table 2**. Summary of the models of the multivariable logistic regression analysis of analytical and radiological parameters on admission according to Clinical Frailty Scale. **Supplementary Table 3**. Summary of the models of the multivariable logistic regression analysis of outcome according to Clinical Frail.

## Data Availability

The datasets generated during the current study are not publicly available due data are not publicly available due to privacy or ethical restrictions, but are available from the corresponding author on reasonable request.

## Abbreviations

BNP: Brain natriuretic peptide
CCI: Charlson comorbidity index
CFS: Clinical frailty scale
COVID-19: Coronavirus disease2019
eGFR: Estimated glomerular filtration rate
ICU: Intensive care unit
IMV: Intensive mechanical ventilation
IQR: Interquartile range
SARS.CoV-2: Severe acute respiratory distress syndrome coronavirus 2
