# Supplementary material for: Association between Clinical Frailty Scale (CFS) and clinical presentation and outcomes in older inpatients with COVID-19
Source: BMC Geriatr. 2023 Jan 2;23:1. doi: 10.1186/s12877-022-03642-y (PMC9806809; doi:10.1186/s12877-022-03642-y)
Supplement: Supplementary file 1 — Additional file 1: Supplementary Table 1. Summary of the models used in the multivariable logistic regression analysis of symptoms according to Clinical Frailty Scale. Supplementary Table 2. Summary of the models of the multivariable logistic regression analysis of analytical and radiological parameters on admission according to Clinical Frailty Scale. Supplementary Table 3. Summary of the models of the multivariable logistic regression analysis of outcome according to Clinical Frail. [file 12877_2022_3642_MOESM1_ESM.docx]

**Supplementary Table 1**. Summary of the models used in the multivariable logistic regression analysis of symptoms according to Clinical Frailty Scale

| **Symptoms according to frailty*** | **Log-likelihood**^†^ | **Cox-Snell R^2‡^** | **Nagelkerke R^2‡^** |
| --- | --- | --- | --- |
| **Duration of symptoms < 5 days prior to admission** | | | |
| Mild-moderate/severe frailty | 980.100 | 0.57 | 0.077 |
| Any frailty | 985.012 | 0.051 | 0.068 |
| **Fever** |  |  |  |
| Mild-moderate/severe frailty | 1015.040 | 0.069 | 0.092 |
| Any frailty | 1055.853 | 0.025 | 0.033 |
| **Dry cough** |  |  |  |
| Mild-moderate/severe frailty | 1038.066 | 0.041 | 0.054 |
| Frailty | 1041.938 | 0.036 | 0.048 |
| **Wet cough** |  |  |  |
| Mild-moderate/severe frailty | 699.804 | 0.009 | 0.015 |
| Frailty | 699.825 | 0.009 | 0.015 |
| **Dyspnea** |  |  |  |
| Mild-moderate/severe frailty | 1045.723 | 0.017 | 0.022 |
| Frailty | 1048.425 | 0.113 | 0.018 |
| **Anosmia-dysgeusia** |  |  |  |
| Mild-moderate/severe frailty | 465.350 | 0.048 | 0.098 |
| Frailty | 467.201 | 0.05 | 0.094 |
| **Myalgia-arthralgia** |  |  |  |
| Mild-moderate/severe frailty | 746.217 | 0.080 | 0.122 |
| Frailty | 748.012 | 0.077 | 0.118 |
| **Asthenia** |  |  |  |
| Mild-moderate/severe frailty | 1027.892 | 0.041 | 0.055 |
| Frailty | 1028.197 | 0.040 | 0.054 |
| **Diarrhea** |  |  |  |
| Mild-moderate/severe frailty | 793.158 | 0.058 | 0.087 |
| Frailty | 798.105 | 0.052 | 0.078 |
| **Confusion** |  |  |  |
| Mild-moderate/severe frailty | 530.462 | 0.081 | 0.151 |
| Frailty | 536.778 | 0.074 | 0.137 |

*Clinical Frailty Scale: 0–4, no frailty; 5–9, any level of frailty; 5–6, mild-moderate frailty; 7–9, severe frailty. Reference value: no frailty, adjusted for age group (65-74, 75-84 and ≥ 85 years), sex, wave of infections, residence in nursing home, and Charlson comorbidity index (dichotomized: 0-4 and ≥ 5)

†The Log-likelihood ensures that the maximum value of the log probability occurs at the same point as the original probability function. The log-likelihood function has many applications, but one is to determine whether one model fits the data better than another.

**^‡^**To evaluate the goodness-of-fit of the logistic regression model, calculating Cox-Snell R^2^ and Nagelkerke R^2^ statistics. Nagelkerke R^2^ is an adjusted version of the Cox-Snell R^2^. Both tests measure the proportion of the total variation of the dependent variable that can be explained by independent variables in the current model. The Cox-Snell R^2^ and Nagelkerke R^2^ expresses the explanatory power of the model.

**Supplementary Table 2**. Summary of the models of the multivariable logistic regression analysis of analytical and radiological parameters on admission according to Clinical Frailty Scale

| **Analytical and radiological variables according to frailty*** | **Log-likelihood**^†^ | **Cox-Snell R^2‡^** | **Nagelkerke R^2‡^** |  |
| --- | --- | --- | --- | --- |
| **Oximetry < 94% at room temperature** | | | | |
| Mild-moderate/severe frailty | 1005.480 | 0.008 | 0.011 |  |
| Any frailty | 1005.485 | 0.008 | 0.011 |  |
| **Lymphocytes <1.0 x 103/L** | | | | |
| Mild-moderate/severe frailty | 1047.757 | 0.024 | 0.032 |  |
| Any frailty | 1047.036 | 0.024 | 0.032 |  |
| **C-reactive protein > 10 mg/dL** | | | | |
| Mild-moderate/severe frailty | 996.661 | 0.031 | 0.042 |  |
| Any frailty | 996.476 | 0.031 | 0.043 |  |
| **Procalcitonin > 0.5 ng/mL** | | | | |
| Mild-moderate/severe frailty | 593.719 | 0.028 | 0.050 |  |
| Any frailty | 585.384 | 0.026 | 0.046 |  |
| **Ferritin > 500 mg/L** | | | | |
| Mild-moderate/severe frailty | 919.826 | 0.088 | 0.118 |  |
| Any frailty | 920.579 | 0.087 | 0.117 |  |
| **Lactate dehydrogenase > 250 U/L** | | | | |
| Mild-moderate/severe frailty | 918.795 | 0.020 | 0.028 |  |
| Any frailty | 918.808 | 0.020 | 0.028 |  |
| **D-dimers > 1 mg/mL** | | | | |
| Mild-moderate/severe frailty | 964.458 | 0.088 | 0.118 |  |
| Any frailty | 964.695 | 0.604 | 0.085 |  |
| **Interleukin 6 >10 pg/mL** | | | | |
| Mild-moderate/severe frailty | 565.354 | 0.028 | 0.044 |  |
| Any frailty | 568.131 | 0.023 | 0.037 |  |
| **Brain natriuretic peptide > 125 pg/mL** | | | | |
| Mild-moderate/severe frailty | 581.357 | 0.127 | 0.208 |  |
| Any frailty | 582.322 | 0.126 | 0.206 |  |
| **Potassium < 3.5 mmol/L** | | | | |
| Mild-moderate/severe frailty | 463.594 | 0.015 | 0.028 |  |
| Any frailty | 465.550 | 0.102 | 0.023 |  |
| **eGFR < 60 ml/min/m2** | | | | |
| Mild-moderate/severe frailty | 963.512 | 0.135 | 0.180 |  |
| Any frailty | 964.255 | 0.123 | 0.179 |  |
| **Troponin T > 14 ng/L** | | | | |
| Mild-moderate/severe frailty | 709.043 | 0.257 | 0.357 |  |
| Any frailty | 712.161 | 0.254 | 0.353 |  |
| **Opacities > 50% of lung surface on X-ray** | | | | |
| Mild-moderate/severe frailty | 852.175 | 0.030 | 0.044 |  |
| Any frailty | 854.860 | 0.026 | 0.039 |  |

*Clinical Frailty Scale: 0–4, no frailty; 5–9, any level of frailty; 5–6, mild-moderate frailty; 7–9, severe frailty. Reference value: no frailty, adjusted for age group (65-74, 75-84 and ≥ 85 years), sex, wave of infections, residence in nursing home, and Charlson comorbidity index (dichotomized: 0-4 and ≥ 5)

†The Log-likelihood ensures that the maximum value of the log probability occurs at the same point as the original probability function. The log-likelihood function has many applications, but one is to determine whether one model fits the data better than another.

**^‡^**To evaluate the goodness-of-fit of the logistic regression model, calculating Cox-Snell R^2^ and Nagelkerke R^2^ statistics. Nagelkerke R^2^ is an adjusted version of the Cox-Snell R^2^. Both tests measure the proportion of the total variation of the dependent variable that can be explained by independent variables in the current model. The Cox-Snell R^2^ and Nagelkerke R^2^ expresses the explanatory power of the model.

**Supplementary Table 3**. Summary of the models of the multivariable logistic regression analysis of outcome according to Clinical Frailty Scale

|  | **Adjusted Model A*** | | | | **Adjusted Model B**^†^ | | | |
| --- | --- | --- | --- | --- | --- | --- | --- | --- |
| **Clinical outcomes according to frailty^‡^** | **Log-likelihood^§^** | **Cox-Snell R^2‖^** | **Nagelkerke R^2‖^** | **Log-likelihood^§^** | | **Cox-Snell R^2‖^** | **Nagelkerke R^2‖^** |  |
| **In-hospital mortality** | | | | | | | | |
| Mild-moderate/severe | 735.518 | 0.118 | 0.180 | 536.896 | | 0.134 | 0.215 |  |
| Any frailty | 751.657 | 0.099 | 0.152 | 552.981 | | 0.112 | 0.180 |  |
| **Admission in intensive care unit** | | | | | | | | |
| Mild-moderate/severe | 532.332 | 0.139 | 0.246 | 396.562 | | 0.170 | 0.308 |  |
| Any frailty | 536.004 | 0.135 | 0.239 | 398.707 | | 0.168 | 0.303 |  |
| **Invasive mechanical ventilation** | | | | | | | | |
| Mild-moderate/severe | 414.215 | 0.112 | 0.234 | 396.562 | | 0.170 | 0.308 |  |
| Any frailty | 420.945 | 0.104 | 0.218 | 318.554 | | 0.123 | 0.263 |  |
| **Readmission** |  |  |  |  | |  |  |  |
| Mild-moderate/severe | 377.720 | 0.002 | 0.053 | 296.602 | | 0.034 | 0.084 |  |
| Any frailty | 378.810 | 0.020 | 0.050 | 300.683 | | 0.27 | 0.068 |  |

^*^ Model A adjusted for age group (65-74, 75-84 and ≥85 years), sex, wave, residence in nursing home, and Charlson comorbidity index (dichotomized 0-4 and ≥5).

^†^Model B adjusted for variables in model A, plus procalcitonin > 0.5 ng/mL, ferritin > 500 mg/L, lactate dehydrogenase > 250 U/L, brain natriuretic peptide > 125 pg/mL D-dimers > 1 mg/mL, estimated glomerular filtration rate < 60 ml/min/m2, troponin T > 14 ng/L

**^‡^**Clinical Frailty Scale: 0–4, no frailty; 5–9, any level of frailty; 5–6, mild-moderate frailty; 7–9, severe frailty. Reference value: no frailty, adjusted for age group (65-74, 75-84 and ≥ 85 years), sex, wave of infections, residence in nursing home, and Charlson comorbidity index (dichotomized: 0-4 and ≥ 5)

**^§^**The Log-likelihood ensures that the maximum value of the log probability occurs at the same point as the original probability function. The log-likelihood function has many applications, but one is to determine whether one model fits the data better than another.

**^‖^**To evaluate the goodness-of-fit of the logistic regression model, calculating Cox-Snell R^2^ and Nagelkerke R^2^ statistics. Nagelkerke R^2^ is an adjusted version of the Cox-Snell R^2^. Both tests measure the proportion of the total variation of the dependent variable that can be explained by independent variables in the current model. The Cox-Snell R^2^ and Nagelkerke R^2^ expresses the explanatory power of the model.
